# Cell Signaling of *Caenorhabditis elegans* in Response to Enterotoxigenic *Escherichia coli* Infection and *Lactobacillus zeae* Protection

**DOI:** 10.3389/fimmu.2018.01745

**Published:** 2018-09-10

**Authors:** Mengzhou Zhou, Xiaozhen Liu, Hai Yu, Xianhua Yin, Shao-Ping Nie, Ming-Yong Xie, Wei Chen, Joshua Gong

**Affiliations:** ^1^School of Food and Biological Engineering, Hubei University of Technology, Wuhan, China; ^2^Guelph Food Research Centre, Agriculture and Agri-Food Canada, Guelph, ON, Canada; ^3^State Key Laboratory of Food Science and Technology/International Joint Laboratory on Food Safety, Jiangnan University, Wuxi, China; ^4^State Key Laboratory of Food Science and Technology, Nanchang University, Nanchang, China

**Keywords:** *Lactobacillus*, enterotoxigenic *Escherichia coli*, *Caenorhabditis elegans*, mitogen-activated protein kinase pathway, DAF/IGF pathway, antimicrobial peptides

## Abstract

Enterotoxigenic *Escherichia coli* (ETEC) infection causes the death of *Caenorhabditis elegans*, which can be prevented by certain *Lactobacillus* isolates. The host response of *C. elegans* to ETEC infection and its regulation by the isolates are, however, largely unclear. This study has revealed that, in agreement with the results of life-span assays, the expression of the genes encoding p38 mitogen-activated protein kinase (MAPK) pathway (*nsy-1, sek-1*, and *pmk-1*), insulin/insulin-like growth factor (DAF/IGF) pathway (*daf-16*), or antimicrobial peptides (*lys-7, spp-1*, and *abf-3*) and other defensing molecules (*abf-2, clec-85*) was upregulated significantly when the wild-type nematode (N2) was subjected to ETEC infection. This upregulation was further enhanced by the pretreatment with *Lactobacillus zeae* LB1, but not with *L. casei* CL11. Mutants defective in the cell signaling of *C. elegans* were either more susceptible (defective in NSY-1, SEK-1, PMK-1, or DAF16) or more resistant (defective in AGE-1, DBL-1, SKN-1, or SOD-3) to ETEC infection compared with the wild-type. Mutants defective in antimicrobial peptides (LYS-7, SPP1, or ABF-3) were also more susceptible. In addition, mutants that are defective in NSY-1, SEK-1, PMK-1, DAF16, ABF-3, LYS-7, or SPP1 showed no response to the protection from *L*. *zeae* LB1. The expression of the genes encoding antimicrobial peptides (*lys-7, spp-1*, and *abf-3*) and other defensing molecules (*abf-2, clec-60*, and *clec-85*) were almost all upregulated in AGE-1- or DBL-1-defective mutant compared with the wild-type, which was further enhanced by the pretreatment of *L. zeae* LB1. The expression of these genes was, however, mostly downregulated in NSY-1- or DAF-16-defective mutant. These results suggest that *L. zeae* LB1 regulates *C. elegans* signaling through the p38 MAPK and DAF/IGF pathways to control the production of antimicrobial peptides and defensing molecules to combat ETEC infection.

## Introduction

Probiotics are defined as “live microorganisms which when administered in adequate amounts confer a health benefit on the host” (1) and have widely been used as a food component to promote human health, including enhancement of host immunity and control of enteric bacterial infection (2, 3). Enterotoxigenic *Escherichia coli* (ETEC) are a pathogen commonly causing diarrhea in humans in addition to pigs (4–6). Previous studies have shown that ETEC infection can be controlled by probiotics (7–9). However, the mechanisms underlying probiotic effects, including host responses, remain largely unknown, which are critical for effective development and application of probiotics.

*Caenorhabditis elegans* is a small free-living soil nematode. The short life-span, clear genetic background, easy for culturing and gene manipulation, the simple microbiota and availability of various mutants of *C*. *elegans* have made the nematode an excellent laboratory animal model for studying bacteria and host interactions (10–12). The usefulness of this laboratory animal model in elucidating the molecular mechanisms of probiotic effects has particularly been highlighted by a recent study that revealed a secreted antigen A (SagA, with peptidoglycan hydrolase activity) from *Enterococcus faecium* with a function to protect *C. elegans* against *Salmonella* pathogenesis by promoting pathogen tolerance in a *tol-1*-dependent manner (13). In the past, a number of bacterial pathogens, such as *Pseudomonas aeruginosa* (10), *Salmonella enterica* (14, 15), *Staphylococcus aureus* (16), and *Enterococcus faecalis* (11) have been assessed for their virulence with the nematode model. In addition, *C. elegans* has increasingly been used for preselecting probiotics for pathogen control (17–20). Interestingly, probiotic bacteria preselected by using *C. elegans* were reported to reduce pig diarrhea, demonstrating a correlation of probiotic effect between the two animals (18). Recently, we determined that isolate *Lactobacillus zeae* LB1 was able to protect *C. elegans* from death caused by ETEC infection, which was mediated by inhibiting ETEC enterotoxin production, rather than by interfering colonization of the pathogen in the worm gut (20). The finding was based on the following observations: (1) the expression of ETEC enterotoxin genes was significantly down-regulated during the infection of *C. elegans* by ETEC, which had been pretreated with isolate LB1; (2) the clone with either *estA* or *estB* (enterotoxin genes) expressed in a non-pathogenic *E. coli* was able to effectively kill the nematode and the killing by the clones could also be prevented by isolate LB1; (3) the same isolate only partially inhibited the gene expression of enterotoxins in ETEC or in the clones *in vitro*. Whether *L. zeae* LB1 can also modulate the host immunity to protect *C. elegans* is unclear and is addressed in the present study.

It is known that *C. elegans* immune defense mechanisms are evolutionarily conserved, including the DAF/insulin-like growth factor (DAF/IGF) pathway, p38 mitogen-activated protein kinase (p38 MAPK) pathway, and transforming growth factor-β (TGF-β) signaling pathway (21–24). The *C. elegans* innate immune response consists of the production of numerous antimicrobial proteins and among these proteins, many of their gene expression are inducible upon pathogen infection (25–27). Moreover, some of these putative antimicrobial genes are regulated by signaling pathways involved in the defense of nematodes and mammals against pathogen infection (22, 24, 28). By investigating the behavior of a wild-type *C. elegans* strain, when exposed to ETEC infection and *Lactobacillus* protection, and corresponding gene expression of key components in the p38 MAPK and DAF/IGF pathways and antimicrobial peptides, followed by assessing various mutants defective in particular components of the pathways or antimicrobial peptides for their behavior, we were able to determine that *L. zeae* LB1 could regulate *C. elegans* cell signaling through the p38 MAPK and DAF/IGF pathways to control the production of antimicrobial peptides and related molecules to combat ETEC infection. The results are presented herein.

## Materials and Methods

### *C. elegans* and Bacteria

*Caenorhabditis elegans* N2 Bristol wild-type and mutants that are defective in *lys-7* (mutant ok1384), *nsy-1* (mutant ag3), *pmk-1* (mutant km25), *sek-1* (mutant ag1), *skn-1* (mutant zu67), *dbl-1* (mutant nk3), *spp-1* (mutant ok2703), *abf-3* (mutant ok3366), *daf-16* (mutant mu86), *age-1* (mutant hx546), *sod-3* (mutant gk235), or *ced-9* (mutant n1950) were obtained from Caenorhabditis Genetics Center, University of Minnesota, Minneapolis, MN, USA. The double mutant defective in both *daf-16 and pmk-1* was kindly provided by Dr. Dennis Kim (Department of biology, Massachusetts Institute of Technology, Boston). *C. elegans* strains were routinely maintained on nematode growth medium (NGM) plates seeded with *E. coli* OP50 using standard procedures (29).

K88^+^ ETEC strain JG280 is a hemolytic *E. coli* of serotype O149: K88 (F4), a porcine isolate possessing the toxin genes of *elt, estA, estB*, and *astA*, and antibiotic resistance to tetracycline, ceftiofur, ampicillin, spectinomycin, apramycin, gentamicin, neomycin, and trimethoprim/sulfonamide (6). The ETEC JG280 was cultured in Luria–Bertani (LB) medium at 37°C for 16 h. Following three washes with M9 medium, 200 µl of cell suspension (10^8^ CFU/ml) was spread on a NGM plate (100 mm in diameter) and dried for 3 h at 22°C before beginning of the assay. *Lactobacillus* isolates (*L. zeae* LB1 and *L. casei* CL11) were originally obtained from the adult chicken or pig intestine (18). Either de Man Rogosa Sharpe broth or agar was used to culture *Lactobacillus* isolates at 37°C for 18–24 h in an anaerobic chamber (Coy Laboratory Products, Grass Lake, MI, USA) with an atmosphere of 85% N_2_, 10% CO_2_, and 5% H_2_. After three washes with M9 medium, 200 µl cell suspension of each *Lactobacillus* isolate (10^8^ CFU/ml) was spread on a NGM plate (100 mm in diameter) and dried for 3 h at 22°C prior to the use.

### Life-Span Assay of *C. elegans*

The life-span assays of *C. elegans* were performed using the methods published previously (17, 20) with some modifications. Briefly, the synchronized *C. elegans* were transferred to NGM agar with *E. coli* OP50 at 25°C for 48–60 h until they reached the L4 stage. In the assay, there were usually three types of a treatment including: (1) Control, (2) ETECT Infection only, and (3) Probiotic Pretreatment. In the control group, the nematode was treated with *E. coli* OP50 (food for *C. elegans*) only throughout the entire assay. In the group of ETECT infection only, the nematode was incubated with *E. coli* OP50 for 18 h followed by incubation with ETEC for up to 15 days in the absence of *E. coli* OP50. In the group of Probiotic Pretreatment, the nematode was incubated with a *Lactobacillus* isolate (either *L. zeae* LB1 or *L. casei* CL11) for 18 h followed by incubation with ETEC for up to 15 days in the absence of *E. coli* OP50. The incubation temperature was 25°C. Each assay was started by transferring L4 stage worms (50 worms per replicate and 3 replicates per treatment) onto the agar plates seeded with either *E. coli* OP50 or a *Lactobacillus* isolate, which was designated as day 0. After 18 h incubation, worms on each plate within the group of either ETEC Infection only or Probiotic Pretreatment were transferred to a fresh NGM plate daily that had been seeded with ETEC JG280 and was subsequently incubated at 25°C. In parallel, worms within the Control group were transferred to a fresh NGM plate daily that had been seeded with *E. coli* OP50 after the 18 h incubation with the same bacterium. The survival of nematode was examined at 24-h intervals for up to 15 days. To determine the survival of *C. elegans*, the number of live worms was recorded daily, and the percentage of surviving worms was calculated by the following formula: survival (%) = (live worms/total worms used) × 100. A worm was considered to be dead when it failed to respond to touch. In the assay where a mutant was examined, the procedure remained unchanged except that the wild-type nematode was replaced by the mutant. Each assay was repeated at least twice unless it is otherwise indicated.

### RNA Extraction

**Figure 1:**
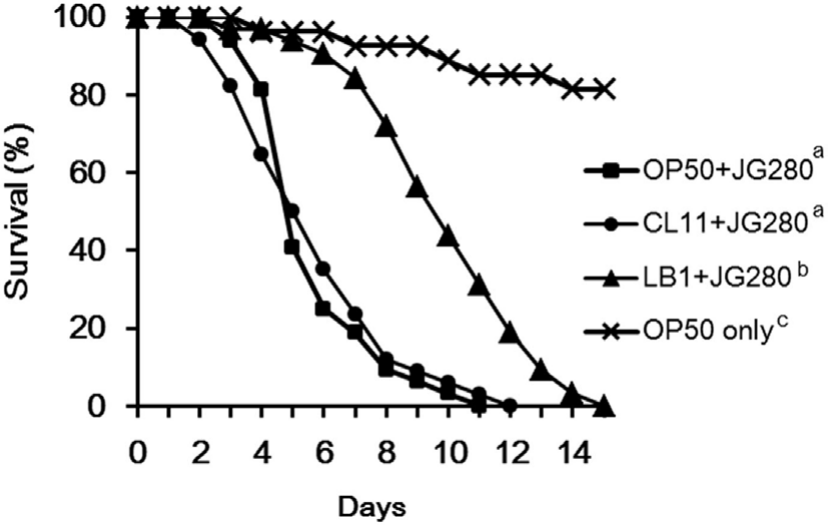
Effects of feeding isolates LB1 (*Lactobacillus zeae*) and CL11 (*Lactobacillus casei*) on the survival of the wild-type *Caenorhabditis elegans* (N2) after infection with Enterotoxigenic Escherichia coli (ETEC) JG280. The worms were first fed either with *E. coli* OP50 or *Lactobacillus* (isolate LB1, CL11) at 10^8^ CFU/ml for 18 h and then ETEC JG280 for the remaining days. Treatments: ■, *E. coli* OP50 and then ETEC JG280; ●, isolate CL11 and then ETEC JG280; ▲, isolate LB1 and then ETEC JG280; and ×, treated with *E. coli* OP50 only. All the groups showing different letters were significantly different (*P* ≤ 0.01) in their survival curves.

To prepare the lysates for RNA extraction, approximately 150 worms from each treatment were sampled on day 2 of the life-span assay. The sampling date were selected based on the observation that the death of worms started to increase on days 3 and 4 after ETEC infection (Figure 1). Following two washes with PBS (pH 7.4), the worms were disrupted in 0.8 ml of a Lysis/Binding Buffer from the mirVana miRNA Isolation Kit (Ambion, TX, USA) by a bead-beater (PowerLzyer24, MO BIO Laboratories, Inc., Carlsbad, CA, USA). The beating was conducted at 3,500 rpm for two cycles followed by four cycles at 3,000 rpm and four cycles at 2,500 rpm. Each cycle lasted for 1.5 min and there was a 2-min interval between two cycles with the samples on ice. The total RNA of *C. elegans* was then extracted using the mirVana miRNA Isolation Kit according to manufacturer’s instructions. After RNA extraction, the samples were treated with DNase I (Ambion, TX, USA) at 37°C for 30 min and then verified as DNA-free by PCR assays with primers specific to *act-1*. RNA integrity was determined by visualization in an agarose gel. The concentration of total RNA was determined with a NanoDrop ND-1000 spectrophotometer (NanoDrop Technologies, Wilmington, DE, USA).

### Reverse Transcription and Real-Time QPCR Analysis

The *C. elegans* gene expression was determined by reverse transcription and quantitative PCR (QPCR) analysis as described previously (30) with some modifications. Briefly, a RNA sample was used for first-strand cDNA synthesis using the SuperScript first-strand synthesis system (Invitrogen, Carlsbad, CA, USA) according to manufacturer’s instructions. Housekeeping genes *snb-1* and *act-1* were used as internal controls for relative quantification of gene expression. QPCR assays were performed using 7500 Real-Time PCR System (Applied Biosystems, Foster, CA, USA) and brilliant SYBR green QPCR master mix (Bio-Rad Laboratories, Richmond, VA, USA). All the primers for PCR assays are listed in Table 1. The primers developed from the present study were designed using the primer designing tool by NCBI (https://www.ncbi.nlm.nih.gov/tools/primer-blast) and verified experimentally by sequencing the amplicons from each pair of the primers (data not shown). In the QPCR assays, 1μl of each cDNA sample was included in a 24-µl reaction mixture containing 12.5 µl Master Mix, 3.75 µl each of the primers at 150 nM, and 4 µl irradiated and double autoclaved dH_2_O. The QPCR program included 5 min at 95°C and 40 cycles of 95°C for 30 s, 56°C for 1 min, and 72°C for 30 s. Fluorescence was measured after each annealing during the cycles.

**Table 1:** Primers of QPCR assay^a^.

Primer	Amplicon (bp)	Sequence (5′–3′)	Source or reference
Act-1-F	121	CCCCACTCAATCCAAAGGCT	This study
Act-1-R		GTACGTCCGGAAGCGTAGAG	
Daf-16-F	181	TCGTCTCGTGTTTCTCCAGC	This study
Daf-16-R		TAATCGGCTTCGACTCCTGC	
Age-1-F	359	CTCCTGAACCGACTGCCAAT	This study
Age-1-R		AAATGCGAGTTCGGAGAGCA	
Lys-7-F	153	GTACAGCGGTGGAGTCACTG	This study
Lys-7-R		GCCTTGAGCACATTTCCAGC	
Clec-60-F	219	CGGTTTCAATGCGGTATGGC	This study
Clec-60-R		TGAAGCTGTGGTTGAGGCAT	
Clec-85-F	121	CCAATGGGATGACGGAACCA	This study
Clec-85-R		CTTCTGTCCAGCCAACGTCT	
Abf-3-F	189	AACAGATTGGGGTCAGCTCG	This study
Abf-3-R		TGGAGACCATTATTGCCGGG	
Spp-1-F	106	TGGACTATGCTGTTGCCGTT	This study
Spp-1-R		ACGCCTTGTCTGGAGAATCC	
Abf-2-F	176	CCGTTCCCTTTTCCTTGCAC	This study
Abf-2-R		GACGACCGCTTCGTTTCTTG	
Tir-1-F	223	TTGGGTGCACAAAGAGCTGA	This study
Tir-1-R		GGTCGGTGTCGTTCTGTTCA	
Nsy-1-F	122	AGCGGCTCGATCAACAAGAA	This study
Nsy-1-R		CCCATTCCACCGATATGCGA	
Sek-1-F	158	CACTGTTTGGCGACGATGAG	This study
Sek-1-R		ATTCCGTCCACGTTGCTGAT	
Pmk-1-F	115	CCAAAAATGACTCGCCGTGA	This study
Pmk-1-R		CTTTTGCAGTTGGACGACGA	
Bar-1-F	119	CATGGTAGTCCGCGACTTGT	This study
Bar-1-R		CGAGAATTGACCAGCTCCAGA	
Skn-1-F	153	CTGGCATCCTCTACCACCAC	This study
Skn-1-R		TTGGTGATGATGGCCGTGTT	
Dbl-1-F	194	TTTTGCGGCGAACAAATCGT	This study
Dbl-1-R		TTCGCTGTTGCCTGTTTGTG	
Snb-1-F		CCGGATAAGACCATCTTGACG	(24)
Snb-1-R		GACGACTTCATCAACCTGAGC	

*^a^All the PCR products amplified with the pairs of primers designed in this study have been verified by DNA sequencing*.

The QPCR data were analyzed using the 2^−ΔΔCt^ method to determine the relative abundance (fold changes) of target genes (31). The cycle threshold, Ct, is the point at which fluorescence above the background is statistically significant. Ct values were determined with the 7500 Real-Time PCR System software based on a threshold line that was manually defined above the non-informative fluorescent data. The ΔCt represents the difference between the Ct value with the primers to a target gene and the Ct value to the housekeeping genes. The ΔΔCt represents the difference between the ΔCt value of treatment group (either treated with *Lactobacillus* or ETEC JG280, or a mutant) and the ΔCt value of control group (treated with *E. coli* OP50, or the wild-type nematode). The values derived from 2^−ΔΔCt^ represent fold changes of samples in abundance relative to the reference samples. The reference samples (either the samples treated with *E. coli* OP50 or the samples of wild-type nematode) had the 2^−ΔΔCt^ value of 1.

### Statistical Analysis

All statistical computation analyses were performed using the Statistical Analysis System (SAS release 9.2, SAS Institute Inc., Cary, NC, USA). Survival curves for *C. elegans* were compared using the Kaplan–Meier survival analysis followed by a log-rank test. One-way analysis of variance and the Tukey’s multiple comparisons were carried out to test for significant differences between the means. Means with *P* values ≤0.05 were considered to differ significantly.

## Results

### Enhancement in the Resistance of *C. elegans* to ETEC JG280 Infection by *L. zeae* LB1

In our previous study, a temperature-sensitive mutant (SS104) of *C. elegans* that harbors a temperature-sensitive allele of *glp-4* (bn2) was used to develop a *C. elegans* infection and protection assay for preselection of probiotics (20). In the present study, identification of the host response of *C. elegans* at the molecular level to the infection of ETEC and also to the protection offered by *Lactobacillus* was the goal. Since there was a need to use various mutants of *C. elegans*, the wild-type nematode (N2) had to be used in the present study in order to compare the behavior of the wild-type and mutants in both life-span assays and gene expression experiments. Figure 1 shows the effects of isolates *L. zeae* LB1 and *L. casei* CL11 on the life-span of *C. elegans* infected with ETEC. The wild-type nematode (N2) pretreated with isolate LB1 had a significantly extended life-span (*P* ≤ 0.05) than those infected with ETEC JG280 only or pretreated with isolate CL11. No significant difference in the life-span (*P* > 0.05) was detected between the worms infected with ETEC JG280 only or pretreated with isolate CL11 followed by ETEC infection. These results are similar to our previously reported observations with a temperature-sensitive mutant (SS104) of *C. elegans* (20).

### Response of Wild-Type *C. elegans* in Gene Expression to ETEC Pretreated With *Lactobacillus* Isolates

**Figure 2:**
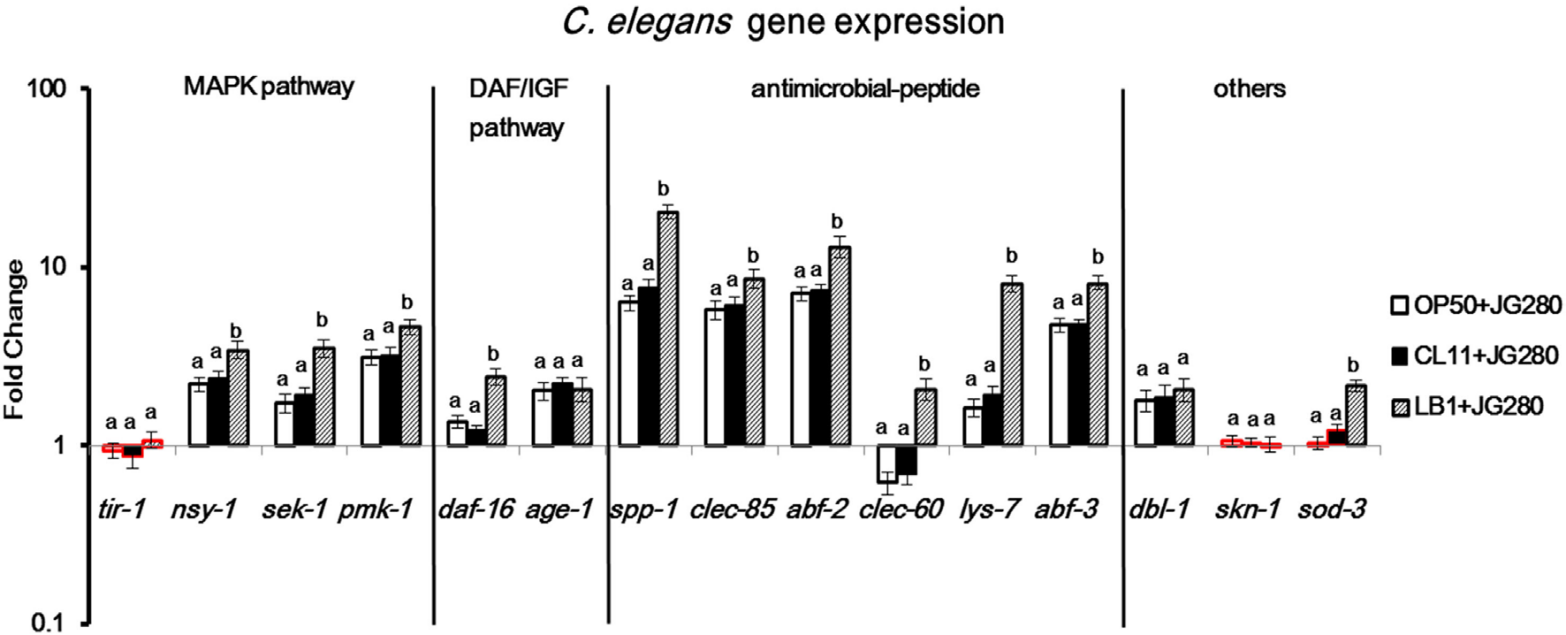
Gene expression of the wild-type *Caenorhabditis elegans* in response to the treatment of Enterotoxigenic *Escherichia coli* (ETEC) JG280 only, or to ETEC JG280 after pretreatment with *Lactobacillus zeae* LB1 or *L. casei* CL11. The nematode was sampled on day 2 of the life-span assays. The baseline is the level of gene expression in *C. elegans* treated with *E. coli* OP50 only. Relative expression was determined using the 2^−ΔΔCt^ method as the ratio of transcript level of each target gene to the housekeeping genes within each group and expressed as fold changes by comparing each treatment group to the control group (*E. coli* OP50). The first ΔCT is the comparison between target genes and housekeeping genes. The second ΔCT represents the comparison between treatment group and control group. Data are presented as mean ± SD. Means marked with “a,” “b” were significantly different (*P* ≤ 0.05) for the same gene among different treatments. While the treatment groups represented by the bars with their borders in red had no significant difference (*P* > 0.05) between each treatment and the control group (fed *E. coli* OP50 only) in the gene expression level of the same gene, the remaining treatment groups represented by the bars with their borders in black differed significantly from the control group (*P* ≤ 0.05).

To examine the host immune response against infection, the wild-type worms on day 2 of the life-span assay were selected since the numbers of viable worms started to dramatically decrease on day 3 and day 4 (Figure 1) and the major components in the p38 MAPK (*tir-1, nsy-1*, sek-1, and pmk-1) and DAF/IGF (*daf-16* and *age-1*) pathways, previously identified antimicrobial peptides (*lys-7, spp-1, abf-2, clec-85, clec-60*, and *abf-3*), and other reported defense molecules (*sod-3, dbl-1*, and *skn-1*) were used as the indicators for the host response and signaling transduction. As shown in Figure 2, when infection with ETEC the expression of almost all selected genes was upregulated (*P* ≤ 0.05) except for *tir-1, clec-60, skn-1*, and *sod-3*. More specifically, expression of the genes associated with the p38 MAPK pathway, such as *nsy-1, sek-1*, and *pmk-1*, or associated with the DAF/IGF pathway, such as *daf-16* and *age-1*, or *dbl-1* gene was all increased significantly (nearly two- to fourfold, *P* ≤ 0.05). Notably, the transcription of antimicrobial peptide, genes such as *lys-7, spp-1, abf-2, clec-85*, and *abf-3* were all upregulated (almost 8–10-fold, *P* ≤ 0.05). Compared with the groups infected with ETEC only, pretreatment of the worms with *L. casei* CL11 showed no effect on the expression of all the selected genes. In contrast, the pretreatment with *L. zeae* LB1 significantly upregulated (*P* ≤ 0.05) the expression of all the selected genes except for *tir-1, age-1, dbl-1*, and *skn-1*.

### Resistance of *C. elegans* to ETEC Infection Involves the p38 MAPK and DAF/IGF Signaling Pathways as Well as Antimicrobial Peptides and Other Related Molecules

**Figure 3:**
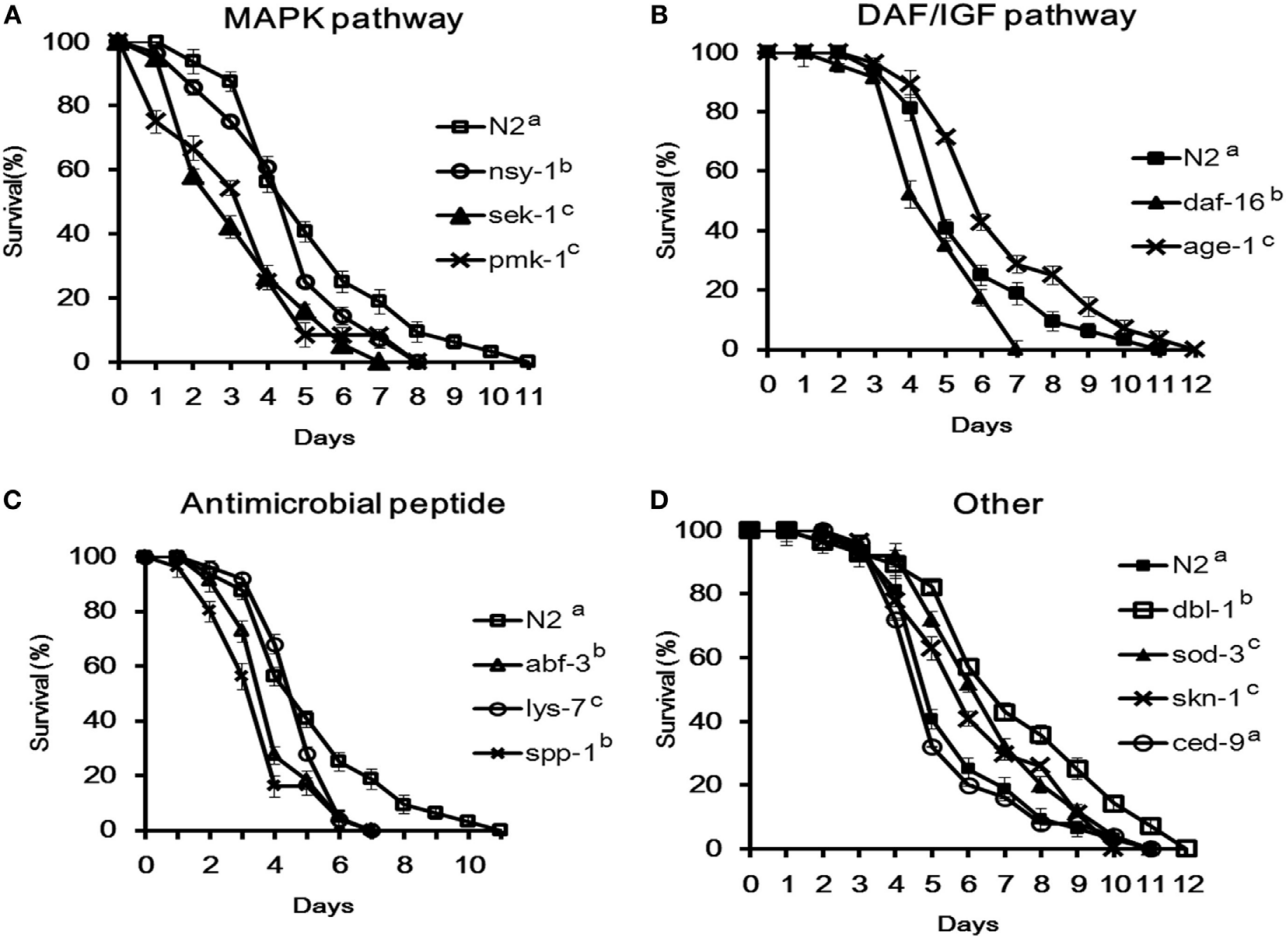
Survival of *Caenorhabditis elegans* mutants infected with Enterotoxigenic *Escherichia coli* (ETEC) JG280. **(A)** Survival curves of the mutants defective in the p38-mitogen-activated protein kinase pathway: *nsy-1(ag3), sek-1(ag1)*, or *pmk-1(km25)*; **(B)** survival curves of the mutants defective in the DAF/IGF pathway: *daf-16(mu86)* or *age-1(hx546)*; **(C)** survival curves of the mutants defective in antimicrobial peptide genes: *lys-7(ok1384), abf-3(ok3366)*, or *spp-1(ok2703)*; **(D)** survival curves of the mutants defective in genes with other functions: *dbl-1(nk3), sod-3(gk235), skn-1(zu67)*, or *ced-9(n1950)*. All the groups were infected with ETEC JG280 after 18 h incubation with *E. coli* OP50 in the life-span assay. The wild-type (N2) served as a reference. All the groups showing different letters were significantly different (*P* ≤ 0.01) in their survival curves.

To determine the roles of *C. elegans* cell signaling in the resistance to ETEC infection, 12 different mutants were used to investigate the life-span of *C. elegans* after ETEC infection. The mutants were divided into four groups: (1) defective in the p38 MAPK pathway including mutants ag3 (defective in *nsy-1*), ag1 (*sek-1)*, and km25 (*pmk-1*); (2) defective in the DAF/IGF pathway including mutants mu86 (*daf-16*) and hx546 (*age-1*); (3) defective in antimicrobial peptides including mutants ok3366 (*abf-3*), ok1384 (*lys-7*), and ok2703 (*spp-1*); and (4) defective in other molecules with a defense function, including mutants nk3 (*dbl-1*), gk235 (*sod-3*), n1950 (*ced-9*), and zu67 (*skn-1*). All the tested three mutants in Group 1 and mutant mu86 (defective in *daf-16)* in Group 2 were highly susceptible to ETEC infection (Figures 3A,B). More specifically, the life-span of these four mutants was reduced by more than 30% compared to the wild-type nematode (N2). Similar results were also observed with the mutants in Group 3 that are defective in antimicrobial peptides (Figure 3C). In contrast, mutant hx546 (defective in *age-1*) in Group 2 and the three mutants (nk3, gk235, and zu67) in Group 4 that are defective in *dbl-1, sod-3*, or *skn-1* were more resistant to ETEC infection (Figures 3B,D). However, mutant n1950 (defective in *ced-9*) showed no changes in the life-span compared to the wild-type (Figure 3D).

### Immunomodulatory Activity of *L. zeae* LB1 Involves Some Components in the p38 MAPK and DAF/IGF Signaling Pathways as Well as Antimicrobial Peptides

**Figure 4:**
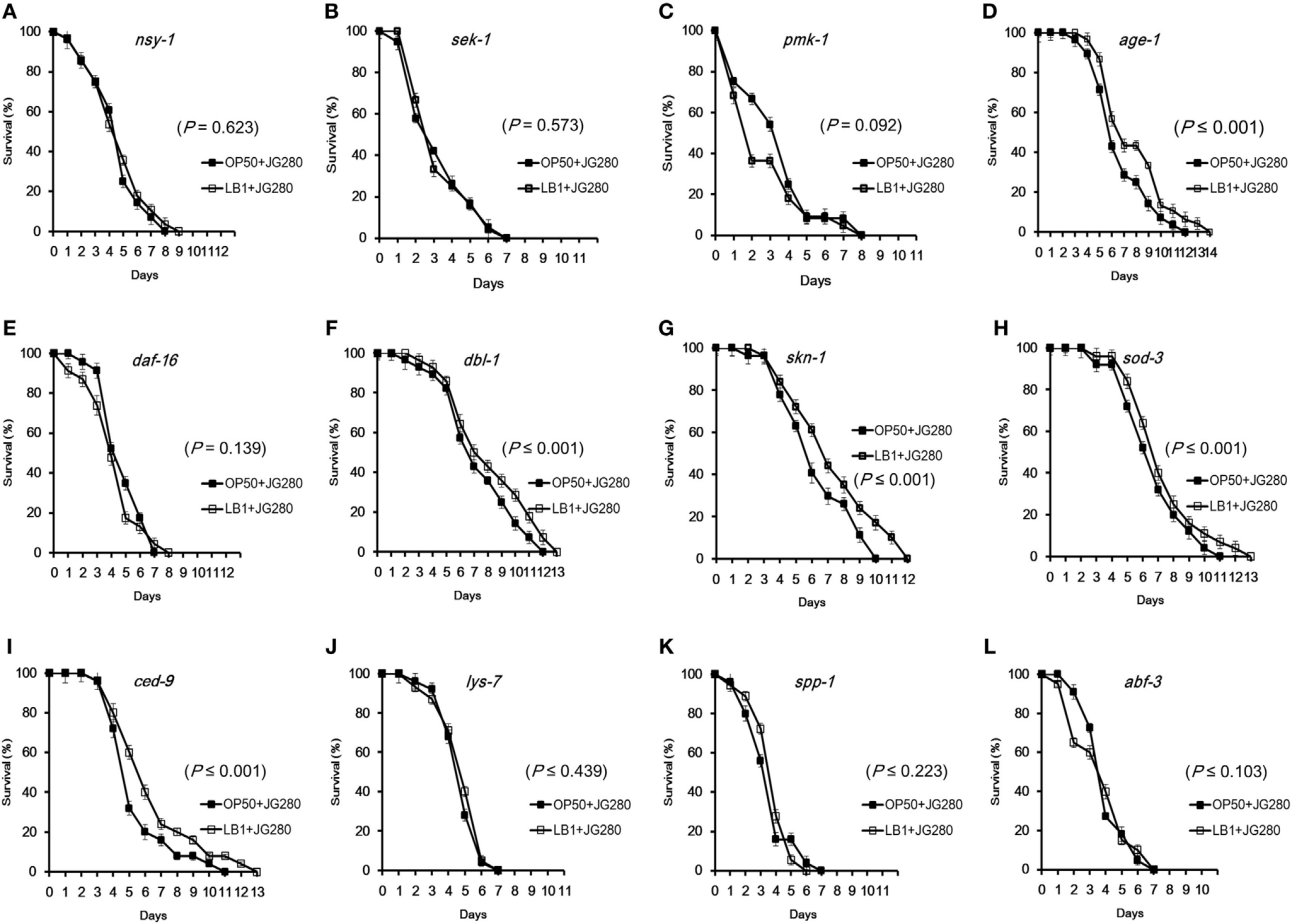
Effects of *Lactobacillus zeae* LB1 on the resistance to Enterotoxigenic *Escherichia coli* (ETEC) infection of *Caenorhabditis elegans* mutants defective in the genes for cell signaling or defense molecules and antimicrobial peptides. The mutants are defective in *nsy-1* **(A)**, *sek-1* **(B)**, *pmk-1* **(C)**, *age-1* **(D)**, *daf-16* **(E)**, *dbl-1* **(F)**, *skn-1* **(G)**, *sod-3* **(H)**, *ced-9* **(I)**, *lys-7* **(J)**, *spp-1* **(K)**, or *abf-3* **(L)**. The life-span assay was conducted in the absence or presence of *L. zeae* LB1 during the pretreatment. All *C. elegans* samples were treated with 10^8^ CFU/ml isolate LB1 or *E. coli* OP50 (Control group) for 18 h and then ETEC JG280 for remaining days. Comparisons were made between the worms pre-exposed to *L. zeae* LB1 (LB1 + JG280) and those exposed to ETEC JG280 only (JG280). The *P* value for each comparison is indicated inside of each panel.

To determine if *L. zeae* LB1 could trigger cell signaling of *C. elegans* to resist ETEC infection, the 12 mutants were also examined for their resistance to ETEC infection after pretreatment with the *Lactobacillus* isolate. Interestingly, only the four mutants that are defective in *nsy-1, sek-1, pmk-1*, or *daf-16* and more susceptible to ETEC infection than the wild-type showed no changes to ETEC infection even they were pretreated with *L. zeae* LB1 (Figures 4A–C,E), indicating no protection from the isolate. Moreover, *L. zeae* LB1 showed no protection to the three mutants defective in antimicrobial peptide genes (*lys-7, spp-1*, or *abf*-*3*), i.e., no changes to ETEC infection regardless of the pretreatment with the *Lactobacillus* isolate (Figures 4J–L). In contrast, pretreatment with *L. zeae* LB1 significantly increased (*P* ≤ 0.05) the life-span of the five mutants (Figures 4D,F–I) that were either more resistant or no change in the resistance compared to the wild-type when treated with ETEC JG280 only.

### Regulation of *C. elegans* in Immunomodulatory Activity by *L. zeae* LB1 Through Cell Signaling

**Figure 5:**
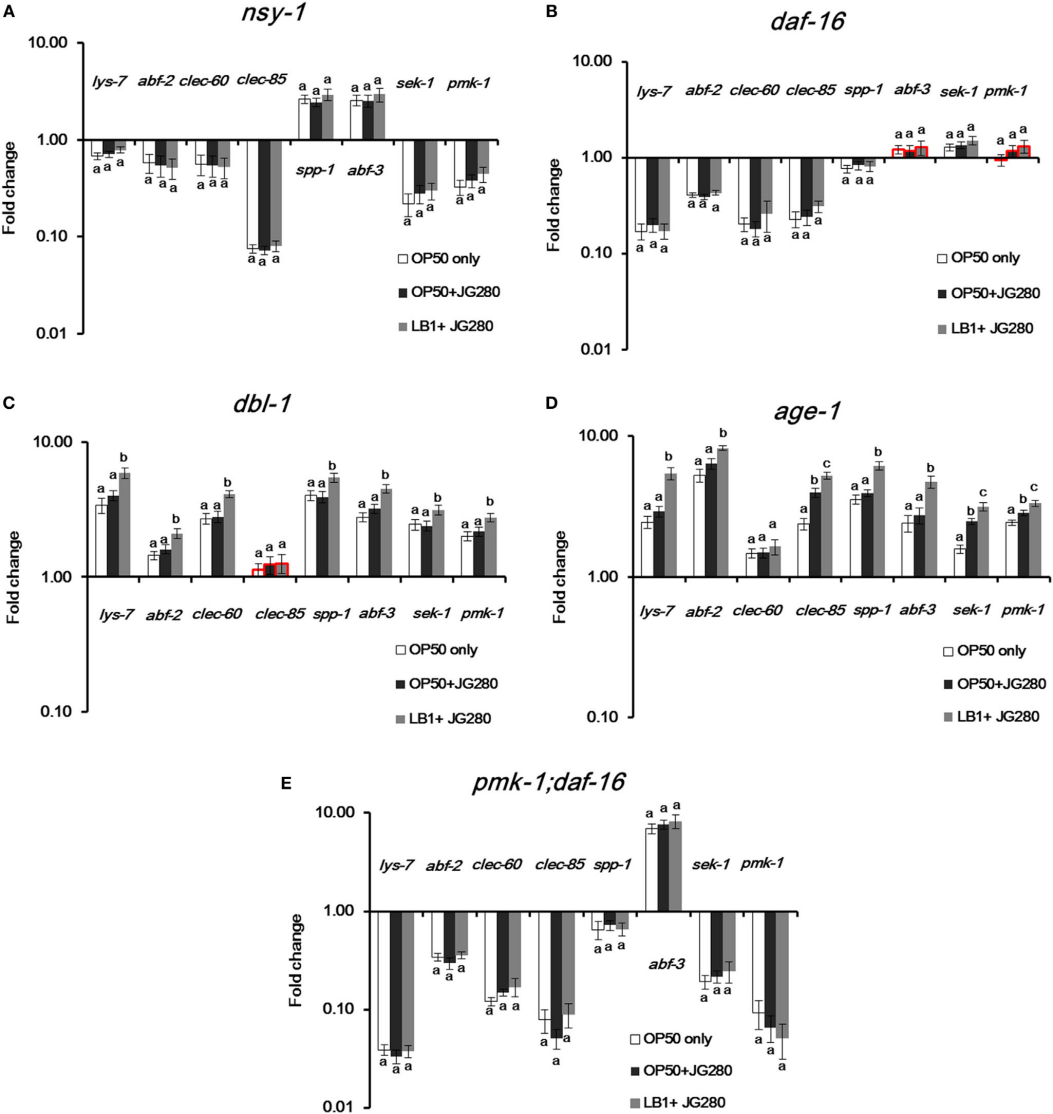
Gene expression of selected *Caenorhabditis elegans* mutants in response to the treatment of *E. coli* OP50, Enterotoxigenic *Escherichia coli* (ETEC) JG280 only, or ETEC JG280 after pretreatment with *Lactobacillus zeae* LB1. The nematode was sampled on day 2 of the life-span assay. The mutants are defective in *dbl-1* **(A)**, *age-1* **(B)**, *nsy-1* **(C)**, *daf-16* **(D)**, or in both d*pmk-1* and *daf-16* **(E)**. The control group is the level of gene expression in *C. elegans* fed OP50 only (open bars). Relative expression was determined using the 2^−ΔΔCt^ method. The first ΔCT is the comparison between target genes and housekeeping genes. The second ΔCT represents the comparison between the mutants and wild-type. The value of each gene in the wild-type is considered to be 1. Means marked with “a,” “b,” and “c” were significantly different (*P* ≤ 0.05) for the same gene among different treatments. While the groups represented by the bars with their borders in red had no significant difference (*P* > 0.05) in the gene expression level of the same gene between each mutant and the wild-type strain (N2) that had been subjected to the same treatment, the remaining groups represented by the bars with their borders in black differed significantly for the same gene within the same treatment when compared to the wild-type (*P* ≤ 0.05).

To elucidate the specific immune response stimulated by *L. zeae* LB1, pretreatment with *L. zeae* LB1 of two ETEC-resistant *C. elegans* mutants that are defective in *age-1* (Figure 3B) or *dbl-1* (Figure 3D), two ETEC-sensitive *C. elegans* mutants that are defective in *nsy-1* (Figure 3A) or *daf-16* (Figure 3B), and one mutant defective in both *pmk-1* and *daf-16* were employed to investigate the regulation of eight selected genes, including *lys-7, spp-*1, *abf-2, clec-85, abf-3, clec-60, sek-1*, and *pmk-1*. The expression of most tested genes, including *lys-7, abf-2, clec-60, clec-85, sek-1*, and *pmk-1* was downregulated significantly (*P* ≤ 0.05) in the mutant defective in *nsy-1* compared to the wild-type, regardless of the treatments (Figure 5A). Similarly, the expression of most tested genes, including *lys-7, abf-2, clec-60, clec-85*, and *spp-1* was also downregulated (*P* ≤ 0.05) in the mutant defective in *daf-16* compared to the wild-type, regardless of the treatments (Figure 5B). A similar trend was also observed in the mutant defective in both *pmk-1* and *daf-16*, in which the expression of all the tested genes except for *abf-3* was downregulated (*P* ≤ 0.05), regardless of the treatments (Figure 5E). On the contrary, the transcription of all the tested genes was all increased substantially (*P* ≤ 0.05) in the mutants either defective in *dbl-1* or *age-1*, except for *clec-60* in the mutant defective in *dbl-1* compared to the wild-type, regardless of the treatments (Figures 5C,D). In view of the responses of all the tested mutants to the three different treatments, i.e., subjected to *E. coli* OP50 only, ETEC infection only, or to the pretreatment with *L. zeae* LB1 before the infection, the three ETEC-sensitive mutants (defective in *nsy-1* or *daf-16*, or double defective in *pmk-1* and *daf-16*) showed no significant differences (*P* > 0.05) in the gene expression within each tested gene (Figures 5A,B,E). In the two ETEC-resistant mutants (defective in *dbl-1* or *age-1*), all the tested genes had no significant differences (*P* > 0.05) in the gene expression between the treatments with *E. coli* OP50 only or with ETEC only (Figures 5C,D). However, the expression of all the tested genes, except for *clec-85* in the mutant defective in *dbl-1* and *clec-60* in the mutant defective *age-1*, was upregulated (*P* ≤ 0.05) by the pretreatment with *L. zeae* LB1.

## Discussion

Although many aspects of innate immunity are shared with higher vertebrates (24), *C. elegans* lacks a cell-mediated immune system and the production of antimicrobial peptides is, therefore, part of its innate immunity to combat bacterial infection (32). The lysozyme (LYS) family, *Ascaris suum* antibacterial factor (ABF) family, SPP (Caenopores are the saposin-like proteins) family, and C-type lectins family are some examples of the nematode innate immunity, which have been shown to play an important role in the general and more specifically induced immune responses to bacterial infection (26, 28, 33). Notably in the present study, the transcription of antimicrobial peptide genes, including *spp-1, clec-85, abf-2, lys-7*, and *abf-3* was significantly upregulated in the wild-type of *C. elegans* in response to ETEC infection (Figure 2), suggesting a vital role of the antimicrobial peptides in the defense of *C. elegans* against ETEC infection. This notion is also supported by the fact that *C. elegans* with a mutation in an antimicrobial peptide gene (*lys-7, spp-1*, or *abf-3*) showed significant shorter life-span than the wild-type when subjected to ETEC infection (Figure 3C). It has been documented from previous studies that the expression of antimicrobial peptide genes in *C. elegans* could be induced by bacterial infection (25, 26, 34–36), which was mainly controlled by the p38 MAPK and DAF/IGF pathways (32, 37). Thus, it can be concluded that antimicrobial peptides play a crucial role in the defense system of *C. elegans* against ETEC infection. Further to this conclusion, there have been reports on specific regulation in the production of antimicrobial peptides by different signaling pathways, e.g., the expression of *lys-1, lys-8, clec-85*, and *nlp-29* through the p38 MAPK pathway (32). In the present study, one new observation was the down regulation in the gene expression of both *abf-2* and *clec-60* in the mutants either defective in *nsy-1* or *daf-16* (Figures 5A,B). In contrast, the expression of *spp-1* was differentially regulated in these two mutants. These results suggest that both *abf-2* and *clec-60* were controlled by both the p38 MAPK and DAF/IGF pathways while *spp-1* was regulated by the DAF/IGF pathway only, which were reported for the first time to the best of our knowledge.

*Caenorhabditis elegans* possesses three major cell signaling pathways in its defense system, including the p38 MAPK, DAF/IGF, and TGF-β pathways (38). The p38 MAPK pathway is the most ancient signal transduction cascade in the nematode immunity, which is mainly associated with antimicrobial responses (39). In parallel, the DAF/IGF signaling pathway transcriptionally regulates many genes involved in the immune and stress responses that is linked to the longevity of *C. elegans* (21, 24). Many candidate antimicrobial genes have been identified in the genome of *C. elegans*; but the role of the signaling pathways in regulating these antimicrobial peptides in response to bacterial infection is yet to be fully elucidated (40–42). In the present study, four genes (*tir-1, nsy-1, sek-1*, and *pmk-1*) in the p38 MAPK pathway, two genes (*daf-16* and *age-1*) in the DAF/IGF pathway, six genes (*lys-7, spp-1, abf-2, clec-85*, and *clec-60*, and *abf-3*) reported previously that encode antimicrobial peptides (27), and three other genes (*sod-3, dbl-1*, and *skn-1*) reported previously with a defense function (23, 43, 44) were initially examined for their possible involvement in the immune response of the wild-type nematode. All the tested genes except for *tir-1, clec-60*, and *skn-1* were upregulated significantly as the results of responding to ETEC infection (Figure 2). While the upregulation suggests the involvement of antimicrobial peptides and defense molecules in the defense system of *C. elegans* as discussed above, it also implies a role of the p38 MAPK and DAF/IGF pathways in regulating the production of antimicrobial peptides and defense molecules. Additional evidence to support the regulatory role of these two pathways is the downregulation of tested genes coding for antimicrobial peptides and defense molecules in the mutants either defective in *nsy-1* or *daf-16* (Figures 5A,B) and in the mutant defective in both *pmk-1* and *daf-16* (Figure 5E). In agreement with these data, the life-span assay indicates that the mutants either defective in *nsy-1, sek-1*, or *pmk-1* (the major components of p38 MAPK pathway) or defective in *daf-16* (a major component of DAF/IGF pathway) were more susceptible to ETEC infection with over 30% reduction in the life-span compared to the wild-type (Figures 3A,B). In view of the data described above, it appears that the regulation of *C. elegans* response in the production of antimicrobial peptides and other molecules with a defense function is mediated mainly through the p38 MAPK and DAF/IGF pathways of cell signaling.

It has been noted from the present study that the regulation in the production of antimicrobial peptides and other molecules with a defense function through the cell signaling pathways could be either positive or negative. This statement is supported by the results from both study of gene expression in the mutants and the life-span assay. In the study of gene expression, tested genes encoding antimicrobial peptides or other molecules with a defense function were upregulated in the mutants either defective in *dbl-1* or *age-1*, but downregulated in the mutants defective either in *nsy-1* or *daf-16* (Figure 5). In agreement with these observations, both mutants either defective in *dbl-1* or *age-1* were more resistant to ETEC infection, whereas the two mutants either defective in *nsy-1* or *daf-16* became more susceptible (Figure 3). These results suggest that genes *dbl-1* and *age-1* have a negative role, while genes *nsy-1* and *daf-16* play a positive role in regulating the production of antimicrobial peptides and defense molecules in the wild-type nematode.

Recently, Kim and Mylonakis (19) reported that the pretreatment of *C. elegans* with *Lactobacillus acidophilus* enhanced the resistance of *C. elegans* to the infection by Gram-positive pathogens *via* the p38 MAPK pathway. Moreover, Bifidobacteria have also been shown to extend the *C. elegans* longevity through the modulation of the p38 MAPK and DAF/IGF pathways (45). Nevertheless, these reports have not yet identified the components in the pathways that *Lactobacillus* or *Bifidobacterium* interacted with to provide protection to the nematode. Very recently, Kamaladevi and Balamurugan (37) reported that *L. case* triggered TLR-mediated RACK-1-dependent p38 MAPK pathway to increase host resistance and protect nematode against *K. pneumoniae* infection. *Bifidobacterium longum* strain BB68 increased the longevity of nematodes by activating the TIR-1—JNK-1—DAF-16 signaling pathway (44). In the present study, the expression of almost all the tested genes encoding antimicrobial peptides or other molecules with a defense function was enhanced significantly by the pretreatment with *L. zeae* LB1 in the mutants that are defective in *dbl-1* or *age-1* when compared to those treated with *E. coli* OP50 or ETEC only (Figures 5C,D). Such enhancement was, however, not detected in the mutants that are defective in *nsy-1* or *daf-16* (Figures 5A,B), or defective in both *pmk-1* and *dal-16* (Figure 5E). Interestingly, the same mutants that are defective in *dbl-1* or *age-1* exhibited longer life-span, whereas the mutants that are defective in *nsy-1* or *daf-16* had shorter life-span than the wild-type (Figure 3). Furthermore, the four mutants that are defective in *nsy-1, sek-1, pmk-1*, or *daf-16* showed no response to the pretreatment with *L. zeae* LB1 and their life-span was similar to the same mutants infected with ETEC only in the life-span assay (Figures 4A–C,E). These data suggest that Nsy-1, Sek-1, Pmk-1, and Daf-16 could be the functional sites in the signaling pathways targeted by *L. zeae* LB1 for the protection.

**Figure 6:**
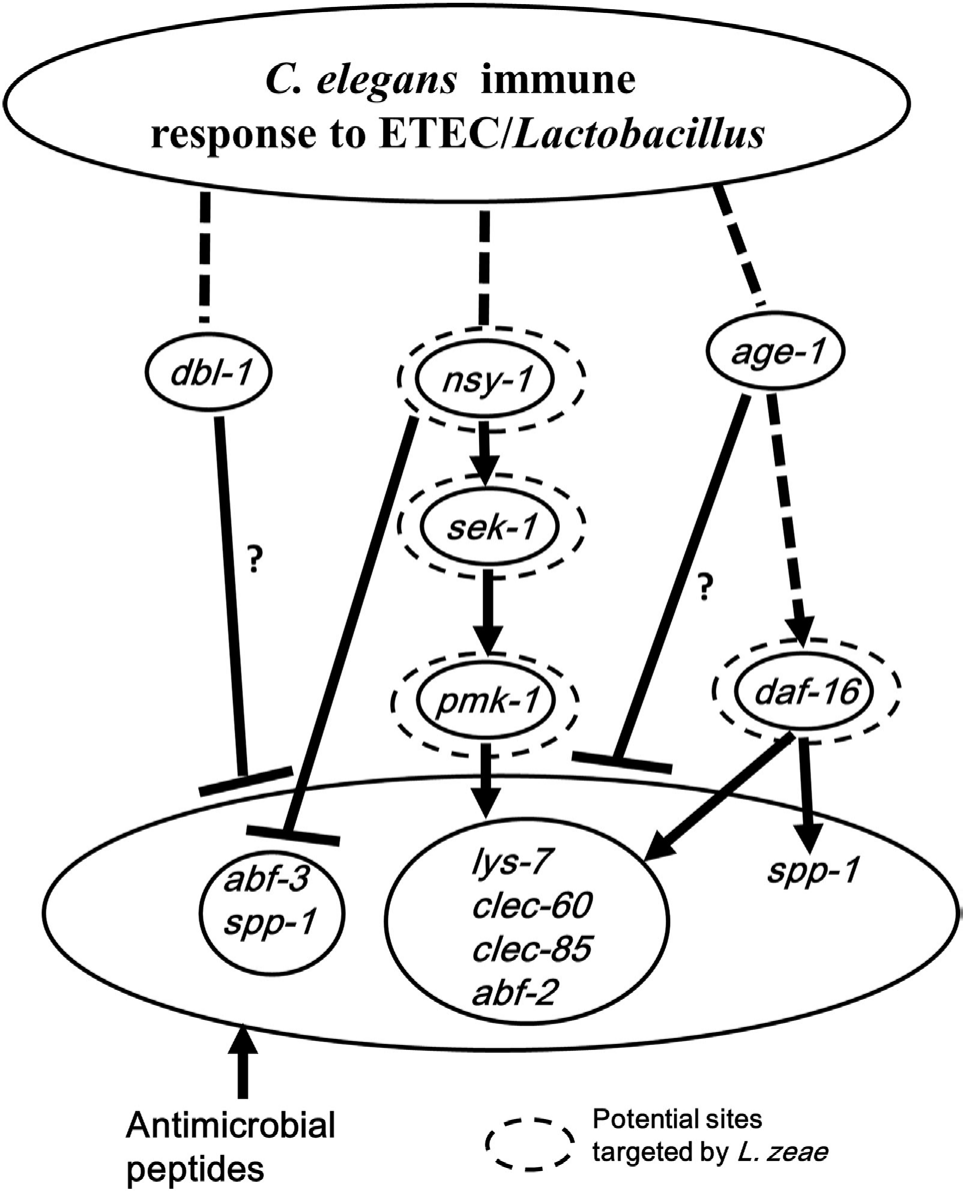
Schematic diagram speculating the immunomodulatory mechanism by *Lactobacillus zeae*. Pretreatment with *L. zeae* LB1 altered the production of antimicrobial peptides through the p38 mitogen-activated protein kinase (MAPK) and DAF/IGF pathways of cell signaling, therefore, changed the resistance of *Caenorhabditis elegans* to Enterotoxigenic *Escherichia coli* (ETEC) infection. The hypothesis was based on the data of both life-span assays and gene expression of various *C. elegans* mutants either pretreated or not treated with *L. zeae* LB1 before the ETEC infection. While dashed lines represent the cascades described in previous literatures, solid lines indicate the effects observed in the present study. ▾, Upregulation; ┴, downregulation. Dashed circle: potential functional sites in the cell signaling targeted by *L. zeae* LB1. ?, Indicating that the regulation could be direct or indirect. More specifically, the downregulation in the production of antimicrobial peptides could be controlled by *dbl-1* or *age-1* directly or through *pmk-1* and *sek-1* in the p38-MAPK pathway in the wild-type nematode (Figures 5C,D).

In conclusion, the present study has revealed: (1) the host response of *C. elegans* to ETEC infection mainly involves both the p38 MAPK and DAF/IGF pathways of cell signaling that regulate the production of antimicrobial peptides and defense molecules; (2) *L. zeae* LB1 alters the production of antimicrobial peptides and other molecules with a defense function through the regulation of both the p38 MAPK and DAF/IGF pathways of cell signaling; (3) gene *nsy-1* or *daf-16* may play a positive role in the regulation, while the role of gene *dbl-1* or *age-1* is negative in the wild-type of *C. elegans*; (4) Nsy-1, Sek-1, Pmk-1, and Daf-16 appear to be the functional sites in the signaling pathways targeted by *L. zeae* LB1 for regulating the production of antimicrobial peptides and other molecules with a defense function. To summarize the new findings from the present study, a schematic diagram has been generated (Figure 6), which also speculates the immunomodulatory mechanism by *L. zeae*.

## Author Contributions

MZ and XL performed the experiments. MZ, HY, XL, and JG analyzed the data. JG, WC, S-PN, and M-YX contributed for reagents/materials/analysis tools. MZ and JG wrote the paper. MZ, HY, XY, and JG designed the experiments. JG and WC conceived the research.

## Conflict of Interest Statement

The authors declare that the research was conducted in the absence of any commercial or financial relationships that could be construed as a potential conflict of interest.
